# Authors’ Reply to Julian Alcazar et al.: “Exploring the Low Force-High Velocity Domain of the Force–Velocity Relationship in Acyclic Lower-Limb Extensions”

**DOI:** 10.1186/s40798-023-00649-6

**Published:** 2023-11-28

**Authors:** Jean Romain Rivière, Jean‑Benoît Morin, Maximilien Bowen, Matt R. Cross, Laurent A. Messonnier, Pierre Samozino

**Affiliations:** 1 Laboratoire Interuniversitaire de Biologie de La Motricité, Univ Savoie Mont Blanc, EA 7424, 73000 Chambéry, France; 2 Laboratoire Interuniversitaire de Biologie de La Motricité, Université Jean Monnet Saint-Etienne, Lyon 1, Université Savoie Mont Blanc, 42023 Saint‑Etienne, France; 3 Sports Performance Research Institute New Zealand (SPRINZ), Auckland University of Technology, Auckland, New Zealand

We appreciate the interest in our article addressing the modeling of the force–velocity relationship in acyclic lower-limb extensions. While we agree with some of the recommendations made, our main disagreement with Alcazar et al. [1] rests on their alternative interpretation of our data, partly due to contrasting philosophies about model selection. Although model selection is a fundamental aspect of the scientific method, it can be challenging and sometimes requires going beyond mere “common sense.”

In the letter [1], a hybrid model that combines linear and hyperbolic functions is reported. This model produces outputs that diverge statistically from the linear model, yet they remain physiologically relevant (i.e., reflecting realistic human performance). The authors suggest the hybrid model is the better of the two to best draws the force–velocity relationship, based on higher “goodness of fit” on the data. If the primary objective is to achieve the highest fit, one might wonder why not adopt more complex models, such as a 10th-order polynomial, which would likely yield a better fit. Indeed, more complex models with greater degrees of freedom (i.e., a higher number of parameters) offer increased flexibility, which can lead to reduced residuals and an enhanced goodness of fit. However, more complex models risk overfitting the data and capturing random biological variability, or noise. The main aim of a model should be to accurately represent a phenomenon without being influenced by the noise. Since there is an equilibrium between overfitting and underfitting, how do we warrant the use of a more complex model? In our view, the answer lies in the principle of parsimony [2].

We invested considerable effort in our paper to describe this equilibrium and to apply the principle of parsimony. Model selection consists of determining which candidate model is the most appropriate considering several performance criteria. For example, in our paper we (i) used common indicators of goodness of fit (e.g., adjusted coefficient of determination and standard error of estimate), (ii) quantified residuals’ distribution across the dependent variable, (iii) sought external physiological validation and discussed reliability of outputs based on other works, and (iv) applied a statistical method to critically analyze each model regarding their degrees of freedom for the sake of parsimony. In comparison, under the pretext of common sense, Alcazar et al. appear to only consider goodness of fit to choose between two models displaying physiologically relevant outputs. As mentioned above, and in our paper, goodness of fit rarely provides a unique criterion for model selection. Furthermore, a significant difference between models’ outputs does not necessarily indicate which model is superior, especially when the true outcome is unknown. In this specific case (i.e., two models with high goodness of fit and different, but physiologically relevant outputs), we recommend selecting the simpler model. By choosing the model with fewer degrees of freedom, we can mitigate risks of overfitting and reduce uncertainty in outputs determination. As suggested by R. McNeil Alexander, “*The simpler the model, the clearer it is which of its characteristics are essential to the observed effect*” [4].

Beyond the selection of performance criteria of a model, the analysis detailed in the letter [1] focuses on the deviation of a single data point. If we follow Alcazar et al.’s recommendation to remove this point based on physiological reasons, it would further validate the appropriateness of the linear model in this context (note we concur with the need for criteria standardization to reduce noise during data collection). Conversely, their analyses were based on data averaged across participants, which does not represent the full range of individual variation within the sample (e.g., individuals presenting no deviation depicted in Fig. 2 of our paper [3]). Individuals presenting the deviation were retained for two reasons. First, the downward deviation in one of the six conditions diverged from the expected value from the linear model by 5%. This difference can likely be attributed to variability in force estimation/measurement (and was magnified when computing power) rather than representing a physiological phenomenon. Second, the slightly low position of this point provided a challenging context for the linear model (i.e., potentially favoring curvilinear models). Consequently, although exclusion of experimental points based on objective criteria deserves further debate, we opted to avoid potential confirmation biases and retain the data point.

In conclusion, we appreciate the opportunity to clarify our perspectives. Asserting that “the force–velocity relationship *is* linear” is untenable, as an absolute truth will never be reached. Nevertheless, even if a model might be considered “wrong”, it can adequately represent reality when based in rigorous analyses. These analyses should strike a balance between the risk of overfitting and the potential of omitting crucial information. In this sense, and beyond the practical advantages in some specific contexts underlined by Alcazar et al. (1), we maintain that the force–velocity relationship of lower limbs in acyclic movements *can be well described* by a linear model. This is because the linear model (i) has displayed physiologically relevant outputs, (ii) aligns with the principle of parsimony, and (iii) consistently yields reliable outputs (see discussion in Rivière et al. [3]), *even if* the data fitting is a bit lower (yet high and sufficient) than with the hybrid model or models with higher degrees of freedom. The hybrid model may yet be the best to describe the external force production capabilities of the lower-limb (neuro)muscular system, but this is based almost entirely on certain fitting performance criteria. In the current context, unless there are compelling reasons—be they physiological rationale or clear experimental evidence—to adopt a more complex model, we think that the simplest model is the best option.

After all, “*Simplicity is the ultimate sophistication*” (L. da Vinci).

## Data Availability

Not applicable.
